# A Colorimetric LAMP Assay for *Salmonella* spp. Detection: Towards a DNA Extraction-Free Approach for Pathogen Screening

**DOI:** 10.3390/foods14030521

**Published:** 2025-02-06

**Authors:** Safae Skenndri, Saâdia Nassik, Rabab Lakhmi, Badr Eddine Anneggah, Fatima Ezzahra Lahkak, Abdeladim Moumen, Imane Abdellaoui Maane

**Affiliations:** 1 Kit and Diagnostic Devices Research Center, MASCIR—Moroccan Foundation for Advanced Science, Innovation and Research, Mohammed VI Polytechnic University, Lot 660, Rabat 10100, Morocco; s.skenndri@iav.ac.ma (S.S.); rabab.lakhmi@gmail.com (R.L.); b.anneggah@mascir.ma (B.E.A.); abdellaoui.iman@gmail.com (I.A.M.); 2 Avian Pathology Unit, Department of Veterinary Pathology and Public Health, Hassan II Institute of Agronomy and Veterinary Medicine, Rabat 6202, Morocco; s.nassik@yahoo.com; 3 Physiology and Therapeutics Unit, Department of Veterinary Biological and Pharmaceutical Sciences, Hassan II Institute of Agronomy and Veterinary Medicine, Rabat 6202, Morocco; f.lahkak@iav.ac.ma

**Keywords:** *Salmonella*, foodborne disease, rapid detection, LAMP colorimetric, pretreatment of sample, extraction free

## Abstract

As of today, bacteriological identification and the molecular approach PCR are considered the gold standards for *Salmonella* spp. detection. However, these methods are time-consuming and costly due to the requirements for enrichment and nucleic acid extraction. In this study, we evaluated the reliability of a developed colorimetric loop-mediated isothermal amplification (cLAMP) assay targeting the *hilA* gene, using Phenol Red as an amplification indicator. Given that Phenol Red is pH-dependent, and to develop an extraction-free test, we evaluated chicken meat pretreatment and thermal treatment. First, we assessed the reliability of this test using a pure culture of *Salmonella* spp. and then in 50 chicken samples pretreated with optimal NaOH concentrations under standardized conditions. Samples representing extreme pH values were artificially contaminated and subjected to DNA extraction and a heat-treatment protocol. Serial dilutions of these products served as templates for LAMP reactions. The assay sensitivity was estimated to be around 3.9 CFU/µL of pure bacterial culture. In contrast, in biological samples, we detected up to 10 CFU/µL using DNA extraction, while heat treatment successfully amplified the initial solution and even some dilutions up to 10^3^ CFU/µL. In conclusion, our cLAMP assay demonstrated good sensitivity and provided clear evidence of its potential for in-field use without relying on prior enrichment steps and DNA extraction.

## 1. Introduction

Foodborne illnesses are a worldwide concern. The World Health Organization estimates that 1 person in 10 falls ill every year, on a worldwide scale, resulting in the death of 420,000 people, with 125,000 deaths for children under five. Diarrheal disease is the most prevalent affliction stemming from spoiled food, with *Salmonella* being one of the main causes [1].

In the African region, *Salmonella* is particularly endemic, especially the non-typhoidal serotypes. *Salmonella* Enteritidis and *Salmonella* Typhimurium are the most prevalent, with Sub-Saharan Africa accounting for around 86% of global mortality associated with non-typhoidal infections. Studies have reported a *Salmonella* prevalence of 6.6% in the Middle East and North Africa, with the highest rates observed in Tunisia (10.2%), Morocco (17.9%), and Sudan (9.2%), while the lowest prevalence was found in Oman (1.2%), Jordan (1.1%), and Palestine (1.2%) [2].

In Morocco, regulatory standards emphasize the strict control of *Salmonella* in chicken-based products to mitigate the risks associated with its consumption. According to the Joint Order of the Minister of Agriculture, Maritime Fisheries, Rural Development, and Water and Forests, and the Minister of Health No. 293-19 of 9 Jumada II 1440 (15 February 2019), establishing the list and limits of authorized microbiological criteria for primary products and food products [3], no *Salmonella* is permitted in 25 g of chicken products, except for mechanically separated meat, where the tolerance is limited to 10 g. These regulations highlight the critical need for precise and efficient detection methods that align with national food safety policies, ensuring the protection of public health and compliance with microbiological criteria.

Naturally, detection methods have been used to identify the bacteria in food samples, animal feed, and environmental samples. The gold standards include the horizontal method for the detection of *Salmonella* spp. in the food production chain (ISO 6579-1:2017) [4] and the PCR-based detection (ISO 20837:2006) [5]. While these methods are reliable and trustworthy, on the downside, they are costly and time-intensive, and they require specialized equipment. Throughout the years, a range of molecular techniques have been developed to match the sensitivity and specificity of standard PCR and culture methods while offering faster and more robust performances. Among these, loop-mediated isothermal amplification (LAMP) has emerged as an ideal approach for detecting foodborne pathogens. LAMP is a rapid, simple, and robust amplification method, with its resilience attributed to the use of the Bst polymerase enzyme, which can tolerate inhibitors commonly found in samples [6,7].

The appeal of LAMP in foodborne pathogen detection is evident in the range of assays successfully developed to identify pathogens such as *Salmonella*, Campylobacter, and Listeria, among others. These assays highlight the potential of LAMP in clinical surveillance and the monitoring of infectious diseases [8,9,10]. One key advantage of LAMP over conventional PCR is the potential for visual detection of amplification using a colorimetric indicator. Various indicators can be integrated into a one-pot LAMP reaction, categorized as either direct or indirect. Indirect indicators, such as Hydroxynaphthol Blue (HNB) and Phenol Red (PR), rely on byproducts of DNA synthesis, while direct indicators, like Malachite Green and Leuco Crystal Violet, depend on sequence-specific intercalants or probes [11,12,13,14].

Although LAMP has gained popularity, it often requires a pre-enrichment or enrichment step, with incubation periods ranging from 1 to 24 h, which increases the assay’s overall duration. In this study, we aimed to develop a colorimetric LAMP (cLAMP) assay for detecting *Salmonella* in food samples without the need for pre-enrichment. We also compared the performance of a heat-treatment approach with DNA extraction, using an in-house colorimetric buffer containing Phenol Red as an amplification indicator.

## 2. Materials and Methods

### 2.1. Experimental Design

**Figure 1:**
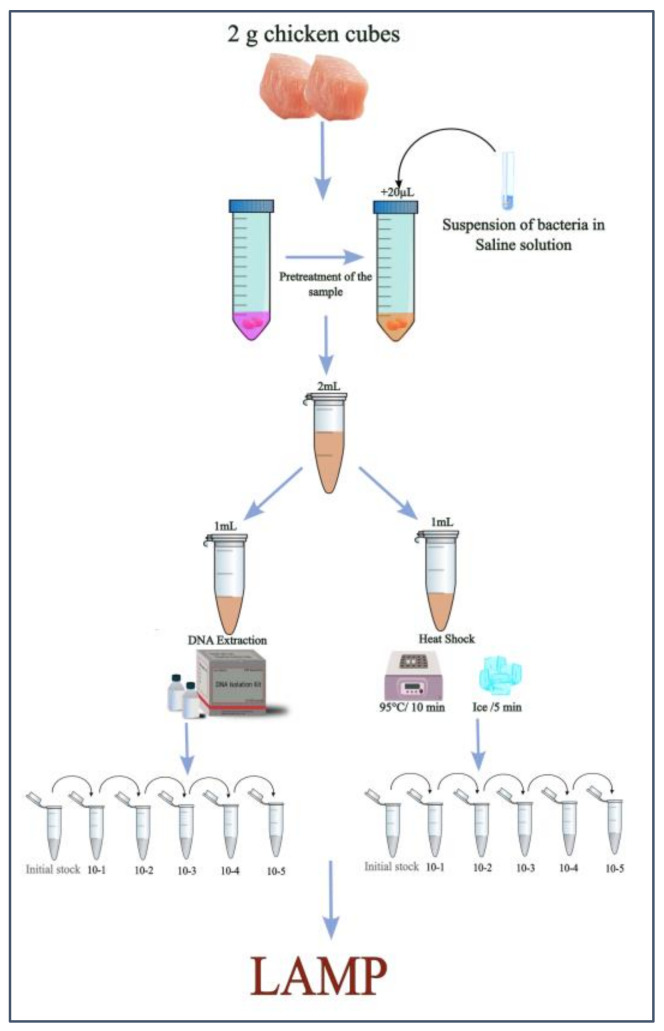
Outline of the experimental design.

To assess the reliability of the cLAMP assay in detecting *Salmonella* spp., a trial was designed (Figure 1). Given that Phenol Red in the reaction buffer is pH-sensitive, a pretreatment protocol was implemented to stabilize the pH of chicken samples. Fifty chicken breast meat samples were pretreated to ensure protocol repeatability. Two of these pretreated samples, spiked with *Salmonella* spp., were selected for further testing.

From each sample, 2 mL of the pretreated product was divided into two portions: one underwent DNA extraction, while the other was processed using a heat-treatment protocol. A 10-fold serial dilution of the DNA extraction and heat-treatment products was then prepared, and these dilutions were used as templates for the cLAMP reactions.

### 2.2. Pretreatment of Chicken Samples and Artificial Contamination

**Figure 2:**
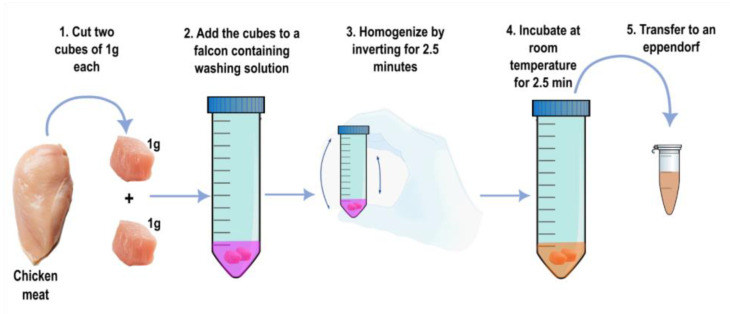
The protocol for pretreatment of chicken samples.

To stabilize the pH of chicken meat samples, multiple parameters were evaluated, including varying NaOH concentrations and volumes, different meat cuts, mixing methods, and incubation times. For each sample, two 1 g cubes of breast meat were prepared and washed with 3 mL of a pretreatment solution containing 12.5 mM NaOH and 0.2 mM Phenol Red (SIGMA-ALDRICH, Saint Louis, MO, USA). The cubes were mixed with the solution by constant inversion for 2.5 min and incubated at room temperature for an additional 2.5 min (Figure 2). Afterward, 2 mL of the pretreatment product was collected, and the pH was measured.

The use of small-sized samples in this study, such as 2 g of meat, was intentional to reduce the overall sample preparation time, the volume of reagents required, and the logistical challenges associated with handling larger sample sizes. This approach aligns with our goal of making the assay more practical for point-of-care applications, where simplicity, speed, and minimal resource use are critical factors.

The contamination solution was prepared using a suspension of *Salmonella* spp. at a concentration of 1.5 × 10^5^ CFU/µL. This strain was recovered from a pure *Salmonella* spp. culture stored in the stock library of the Avian Pathology Unit at the Hassan II Institute of Agronomy and Veterinary Medicine. The solution was centrifuged at 18,000× *g* for 5 min, and the resulting pellet was resuspended in 400 µL of deionized water.

Following a descriptive analysis of pH measurements from 50 pretreated samples, 2 representative samples were selected: one from the first quartile (Q1 = 7.39, Sample A [pH: 7.4]) and another from the third quartile (Q3 = 7.62, Sample B [pH: 7.6]). These samples were then contaminated with 20 µL of the prepared contamination solution and used in the subsequent phase of the experiment: the cLAMP assay.

### 2.3. DNA Extraction

DNA extraction was performed using the PureLink™ Genomic DNA Mini Kit (Invitrogen, Thermo Fisher Scientific, Carlsbad, CA, USA). To prevent interference with the color shift in the LAMP assay, the elution step was carried out using DEPC-treated water (Figure 1). DNA extraction was conducted on both pure *Salmonella* cultures to assess the assay’s sensitivity (in terms of CFU/µL) and on 1 mL of each contaminated sample. The extracted DNA from each sample was then subjected to a 10-fold serial dilution, ranging from 10^−1^ to 10^−5^.

### 2.4. Heat-Treatment Protocol

One milliliter of each sample was heated at 95 °C for 10 min and then immediately chilled on ice for 5 min. The coagulated artifacts formed during the process were allowed to settle for 2 min to simplify sample collection. The heat-treated products were subsequently subjected to a 10-fold serial dilution, ranging from 10^−1^ to 10^−5^.

### 2.5. cLAMP Assay

#### 2.5.1. In-House Colorimetric Buffer Composition

The buffer used in the LAMP reactions contained Phenol Red (354.38 g/mol) at 50 mM, Tris-HCl (157.56 g/mol) (Millipore, Darmstadt, Germany) at 40 mM, KCl (74.551 g/mol) at 1 M, (NH_4_)_2_SO_4_ (132.14 g/mol) at 200 mM, and MgSO_4_ (246.47 g/mol) at 160 mM (OXFORD LAB FINE CHEM LLP, Palghar, India). The pH of the buffer was adjusted to 8.4.

#### 2.5.2. Primer Design and Synthesis

To design the primers for the LAMP assay, a conserved genetic footprint of *Salmonella* enterica subsp. enterica, encompassing all serovars, was selected. The target genetic marker was the *hilA* gene, which encodes a regulatory protein critical for the expression of invasion-related genes essential to the bacterium’s ability to penetrate host cells [15,16]. The primers specific to the *hilA* gene were designed using the PrimerExplorer V5 Primer Design Tool (version 1.4.1) (Table 1). Primers were synthesized by GenScript Biotech Corp., Piscataway, NJ , USA.

#### 2.5.3. cLAMP Sensitivity

The sensitivity of the assay was assessed using a series of 10-fold serial dilutions of a bacterial solution with an initial concentration of 3.9 × 10^4^ CFU/µL. The dilutions, spanning from 3.9 × 10^4^ CFU/µL to 0.39 CFU/µL, were used to determine the lowest detectable concentration of the assay.

#### 2.5.4. cLAMP Specificity

To evaluate the specificity of the cLAMP assay, the DNA extracted from various bacterial strains preserved at the Avian Pathology Unit of the Hassan II Institute of Agronomy and Veterinary Medicine was used as templates. The target bacterial species included *Salmonella* Typhimurium, *Salmonella* Enteritidis, *Salmonella* Gallinarum Pullorum, and *Salmonella* Pullorum. The negative control bacterial species tested were *Escherichia coli*, *Proteus* spp., *Clostridium perfringens*, *Staphylococcus* spp., *Listeria monocytogenes*, *Pasteurella multocida*, *Klebsiella* spp., and *Bordetella* spp.

#### 2.5.5. cLAMP Reaction Mix

The cLAMP assay was performed in a final reaction volume of 25 µL. Each reaction contained 12.5 µL of an in-house colorimetric buffer, 1 µL of Bst 2.0 WarmStart^®^, Ipswich, MA, USA. DNA polymerase (8 U), 3.5 µL of dNTPs (1.6 mM each), 2.5 µL of primer mix (1.6 µM FIP/BIP, 0.2 µM F3/B3, 0.4 µM LF/LB), and 2.5 µL of template (either extracted genomic DNA or heat-treatment product). The LAMP reactions were incubated at 65 °C for 40 min on a heating block using 0.2 mL microtubes. After amplification, the tubes were chilled on ice for 1 min. Amplification was visually detected by a distinct color change from the initial pink to bright yellow, indicating a positive reaction.

### 2.6. Amplification Confirmation

To confirm amplification in the cLAMP reactions, a 1.2% agarose gel electrophoresis was performed. The GelPilot 1kb Plus Ladder (Invitrogen-ThermoFisher Scientific, Vilnius, Lithuania) was used as a molecular weight marker. For each cLAMP product, a 6 µL aliquot was combined with 2 µL of 10X BlueJuice Gel Loading Buffer (Invitrogen, ThermoFisher Scientific, Vilnius, Lithuania) and 2 µL of deionized water. A total of 10 µL of the prepared mixture was loaded into each well. Electrophoresis was conducted at 100 V for 40 min.

### 2.7. Real-Time PCR

To evaluate the performance of the cLAMP assay, the same *Salmonella* solutions used for assessing cLAMP sensitivity were tested in a real-time PCR assay. For each reaction, 2 µL of template was added to a reaction mix containing 2.5 µL of TaqMan Fast Virus 1-Step Master Mix, 0.5 µL of EvaGreen Dye (20X), and 1 µL of a primer mix (forward primer: 5′-GCTACGCTCAGAAAAGAAAGTC-3′; reverse primer: 5′-GTAACTTTGTGACATGCCTGTC-3′), with DEPC-treated water added to reach a final reaction volume of 10 µL. The reactions were run on a QuantStudio™ 6 Flex System using the following thermal cycling protocol: an initial step at 95 °C for 20 s, followed by 40 cycles of 95 °C for 3 s and 60 °C for 30 s.

## 3. Results

### 3.1. Pretreatment of Chicken Samples

To use the cLAMP directly without resorting to DNA extraction, we had to take into consideration the pH of the samples as the assay is pH-dependent since we employed Phenol Red. Normally, the pH of chicken breast meat is acidic (6.5–5.5), and if used directly, it would cause a shift in the color of the reaction mix, exhibiting a yellow color before the start of the amplification in contrast to the desired pink color usually displayed at the beginning. In order to avoid the problem caused by the initial pH of the samples, we decided to stabilize the chicken meat samples using a solution containing NaOH to neutralize the pH into an acceptable range so that the reaction mix would maintain its pink coloration. Multiple NaOH concentrations were screened to find the appropriate one allowing for the stabilization of the pH of the samples (Table 2).

**Figure 3:**
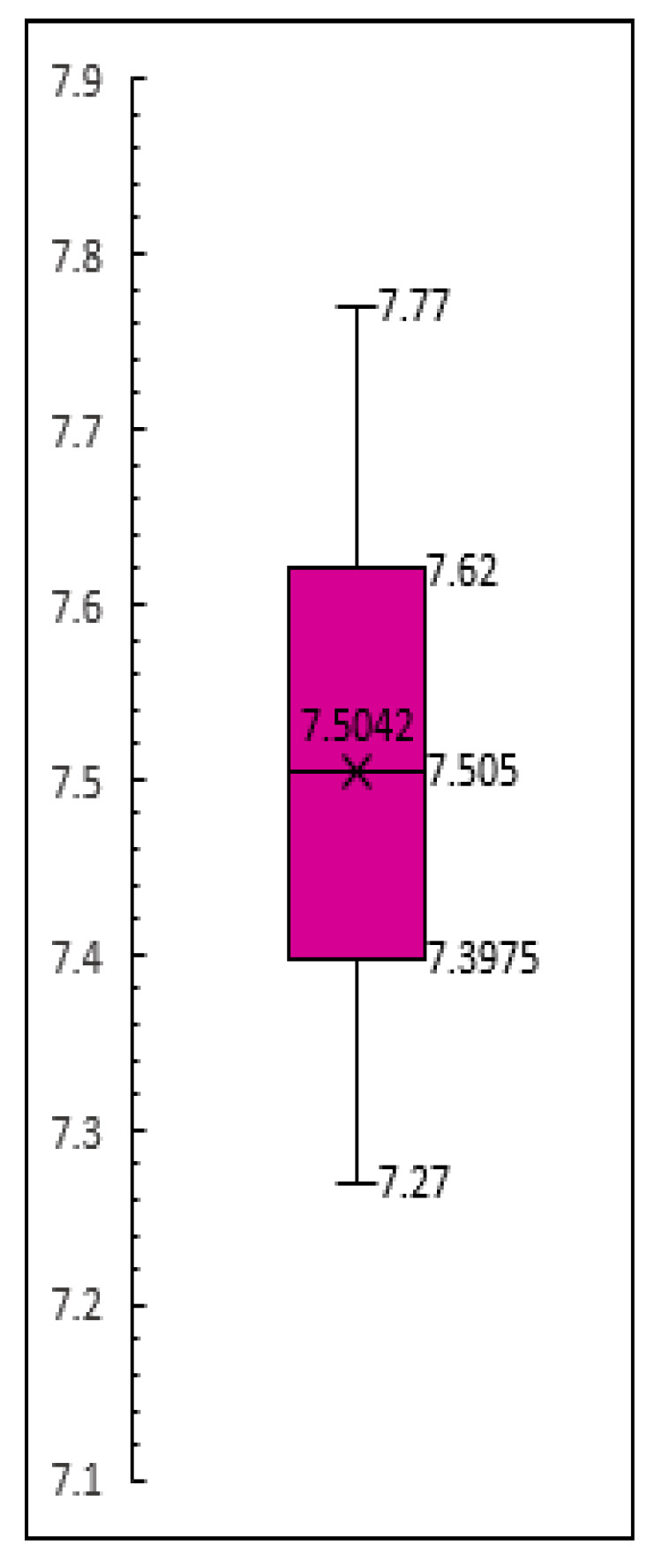
Box plot displaying the distribution of pretreated chicken samples’ pH.

Experiments conducted in our previous studies have revealed that the optimum pH range for a sample that would allow for a smooth amplification of cLAMP assay varied between 7.7 and 7.2, thus, upon screening multiple NaOH concentrations we concluded that 12.5 mM was the best suited for the pretreatment of the chicken meat samples. To ensure the repeatability of the pretreatment protocol, fifty chicken samples were pretreated with the 12.5 mM solution, and the pH was measured. We relied on descriptive statistics to provide insight into the central tendency, dispersion, and other essential characteristics of the dataset. The pH of the pretreated chicken meat samples varied between a minimum of 7.27 and a maximum of 7.77, with a standard deviation of 0.12, indicating that the values of the dataset clustered around the mean (7.5) and were relatively consistent. The absence of outliers was also noted, as visualized on the box plot (Figure 3).

### 3.2. Sensitivity of the Colorimetric LAMP Assay

**Figure 4:**
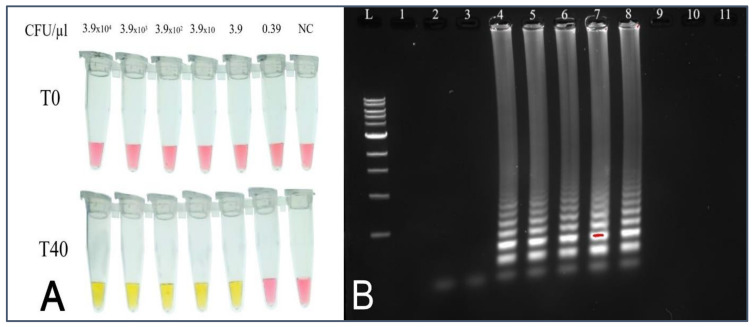
(**A**) Lamp sensitivity test using *hilA* dilution of pure bacterial culture; (**B**) confirmation of amplification of the sensitivity cLAMP reactions. L: ladder 100 bp, 1: void, 2: negative control, 3: 0.39 CFU/µL, 4: 3.9 CFU/µL, 5: 3.9 × 10 CFU/µL, 6: 3.9 × 10^2^ CFU/µL, 7: 3.9 × 10^3^ CFU/µL, and 8: 3.9 × 10^4^ CFU/µL, 9–11: Void.

The assay’s sensitivity was tested using a bacterial solution starting at 3.9 × 10^4^ CFU/µL. Briefly, 10-fold serial dilutions were made, ranging from 3.9 × 10^4^ CFU/µL to 0.39 CFU/µL, to assess the sensitivity. The assay was run at a temperature of 65 °C for 40 min. Therefore, out of the six dilutions of the bacterial solutions used with LAMP reactions, the first five reaction tubes displayed a bright yellow color after 40 min, indicating successful amplification, which was confirmed by gel electrophoresis (Figure 4). The lowest concentration amplified was 3.9 CFU/µL.

### 3.3. Comparison with Real-Time PCR

**Figure 5:**
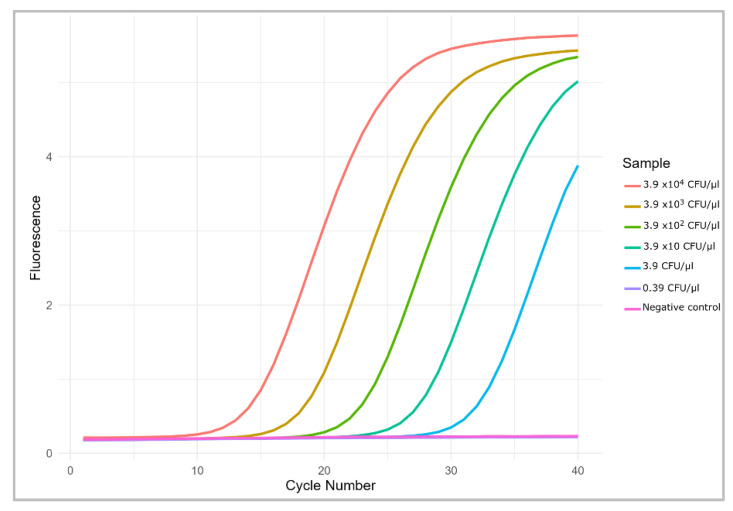
Amplification plot of extracted DNA from serial dilution of *Salmonella* solutions.

The same bacterial solutions used to evaluate cLAMP sensitivity were also utilized for real-time PCR amplification. The lowest concentration successfully amplified using both methods was 3.9 CFU/µL, demonstrating that the cLAMP assay exhibited sensitivity comparable to that of real-time PCR (Figure 5).

### 3.4. cLAMP Specificity Results

**Figure 6:**
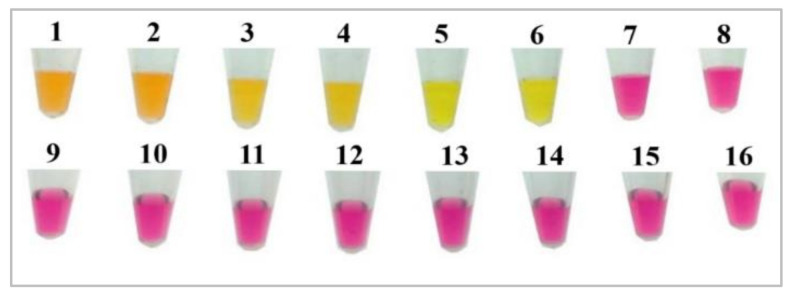
Specificity results of the cLAMP method for *Salmonella* detection. 1: *S*. Enteritidis, 2: *S*. Typhimurium, 3: *S*. Kentucky, 4: *S*. Gallinarum Pullorum, 5–6: positive controls, 7–8: negative controls, 9: *E. coli*, 10: *Proteus mirabillis*, 11: *Klebsiella* spp., 12: *Listeria monocytogenes*, 13: *Pasteurella* spp., 14: *Bordetella* spp., 15: *Staphylococcus* spp., and 16: *Clostridium perfringens*.

To evaluate the specificity of our assay, we tested it against both *Salmonella* and non-*Salmonella* strains. Positive reactions were observed exclusively in tubes containing *Salmonella* serotypes, while no color change was observed in tubes with non-*Salmonella* species. This outcome demonstrates the strong specificity of the assay (Figure 6).

### 3.5. Application of the cLAMP with Artificially Contaminated Samples

#### 3.5.1. cLAMP with Extracted DNA

To evaluate the efficiency of the cLAMP in amplifying the genetic material extracted from contaminated and pretreated samples, 1 mL of the pretreated solution from Sample A [pH: 7.4] and Sample B [pH: 7.6] was used in DNA extraction. The extracted product as well as the five dilutions (1:10) were used as templates for the cLAMP reactions.

**Figure 7:**
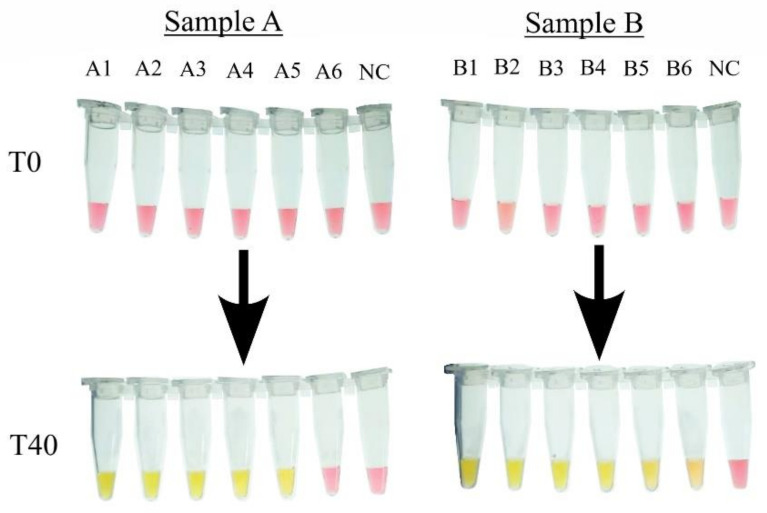
Detection of *Salmonella* in artificially contaminated chicken samples in cLAMP with extracted DNA as template. The number of CFU per µL in each reaction tube, NC : Negative Control, A1: 10^5^ CFU/µL, A2: 10^4^ CFU/µL, A3: 10^3^ CFU/µL, A4: 10^2^ CFU/µL, A5: 10 CFU/µL, A6: 1 CFU/µL; B1: 10^5^ CFU/µL, B2: 10^4^ CFU/µL, B3: 10^3^ CFU/µL, B4: 10^2^ CFU/µL, B5: 10 CFU/µL, and B6: 1 CFU/µL.

For Sample A, a yellow color shift was noted in all reaction tubes except for the last one (A6); the last reaction where amplification was noted contained 10 CFU/µL, as opposed to Sample B, where amplification was noted in all the reaction tubes, with the last reaction containing 1 CFU/µL (Figure 7).

#### 3.5.2. cLAMP with Heat-Treatment Products

While DNA extraction ensures good yield and the purity of the DNA, it is costly, time-consuming, and not compatible with on-field use, all of which highlight the need to swap DNA extraction with another approach that would allow for easy genetic material retrieval while being cost-friendly and suited for on-field use, such as in the heat-treatment method, which relies on the use of a high temperature to lyse the bacterial cell and liberate the genetic material. One of our main objectives in this experiment is to evaluate the efficiency and compatibility of heat treatment with the cLAMP reaction.

**Figure 8:**
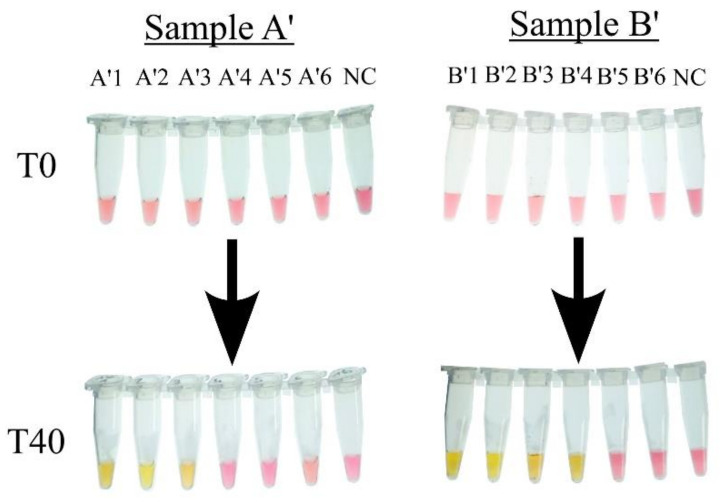
Detection of *Salmonella* in artificially contaminated chicken samples in cLAMP with heat-treatment product as template. The number of CFU per µL in each reaction tube, NC: Negative Control, A’1: 10^5^ CFU/µL, A’2: 10^4^ CFU/µL, A’3: 10^3^ CFU/µL, A’4: 10^2^ CFU/µL, A’5: 10 CFU/µL, A’6: 1 CFU/µL; B’1: 10^5^ CFU/µL, B’2: 10^4^ CFU/µL, B’3: 10^3^ CFU/µL, B’4: 10^2^ CFU/µL, B’5: 10 CFU/µL, and B’6: 1 CFU/µL.

As shown in Figure 8, using the heat-treatment products as templates for the LAMP reactions allowed for the amplification of the first three dilutions for Sample A (A1’, A2’, and A3’), with the last reaction containing 10^3^ CFU/µL, and the first four dilutions in Sample B (B1’, B2’, B3’, and B4’), with the last one containing 10^2^ CFU/µL.

#### 3.5.3. Confirmation of Results with Gel Electrophoresis

**Figure 9:**
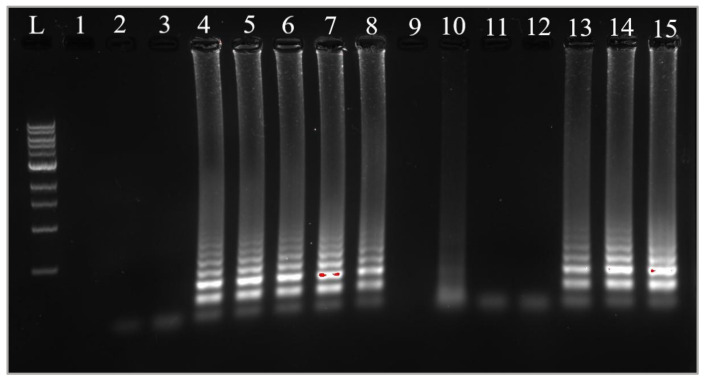
Amplification confirmation by gel electrophoresis for Sample A. L: ladder 100 bp. 2–8: reactions with extracted DNA as template; 10–15 reactions with heat-treatment products as templates; 1: void, 2: NC, 3: A6, 4: A5, 5: A4, 6: A3, 7: A2, 8: A1, 9: void, 10: A’6, 11: A’5, 12: A’4, 13: A’3, 14: A’2, and 15: A’1.

**Figure 10:**
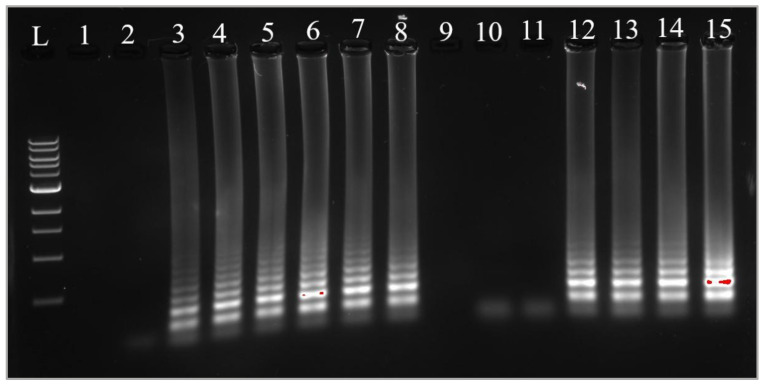
Amplification confirmation by gel electrophoresis for Sample B. L: ladder 100 bp. 2–8: reactions with extracted DNA as template; 10–15 reactions with heat-treatment products as templates; 1: void, 2: NC, 3: B6, 4: B5, 5: B4, 6: B3, 7: B2, 8: B1, 9: void, 10: B’6, 11: B’5, 12: B’4, 13: B’3, 14: B’2, and 15: B’1.

To confirm the amplification occurring in the cLAMP reactions, gel electrophoresis was employed. The results indicated that amplification was successful in the tubes displaying a yellow color, whereas the tubes exhibiting a pink color did not show any amplification in either of the samples. Figure 9 presents a gel image illustrating the migration of amplification products, with extraction products visible in lanes 2 to 8 and heat-treatment products in lanes 10 to 15. Similarly, for Sample B, Figure 10 depicts the extraction products in lanes 2 to 8 and the heat-treatment products in lanes 10 to 15.

## 4. Discussion

This study aimed to develop a robust and reliable detection assay based on the LAMP method, designed to work effectively with biological matrices without requiring traditional DNA extraction. By eliminating the need for commercial extraction kits, the assay becomes more cost-effective, field-friendly, and efficient. Before adapting the assay for use with diverse biological samples, we focused on critical aspects of its design, including primer selection, the development of an in-house colorimetric buffer, and a pretreatment protocol for sample stabilization.

In *Salmonella* detection, the most frequently targeted genetic marker is the invasion gene (invA). However, the bioinformatic analysis of invA revealed multiple single nucleotide polymorphisms (SNPs) among strains, potentially reducing primer sensitivity, particularly given LAMP’s reliance on four to six primers for effective amplification. To overcome this, we selected the *hilA* gene, located in *Salmonella* Pathogenicity Island I, as the target for primer design. This gene encodes a regulatory protein involved in activating the Type III Secretion System, which is essential for host cell invasion. The *hilA* gene is highly conserved across *Salmonella* enterica subspecies enterica serovars, with minimal mutations or SNPs, making it an ideal candidate for assay development. Previous studies targeting *hilA* have demonstrated both high specificity and sensitivity [16,17].

An additional critical component of this assay is the in-house colorimetric buffer, which uses Phenol Red as a pH-dependent indicator. This compound transitions from red (alkaline) to yellow (acidic) during amplification, as pyrophosphate ions are released from DNA polymerization. We optimized this buffer to provide a clear and objective visualization of amplification, with initial conditions set at pH 8.4 to ensure stability throughout the reaction.

The optimized cLAMP assay demonstrated high sensitivity, with a detectable threshold of 3.9 CFU/µL, comparable to that of real-time PCR. For example, studies such as Zendrini et al. (2021) also used Phenol Red-based cLAMP assays to detect up to 10 CFU/g of *Salmonella* without pre-enrichment, showcasing the robustness of such methods [9]. The cLAMP assay also demonstrated high specificity for *Salmonella* species, with no amplification observed for negative control bacterial species.

To enable the direct application of the cLAMP assay without DNA extraction, stabilizing the pH of chicken samples was crucial. Chicken meat, with a native pH of 5.5–6.5, poses a challenge because the assay operates optimally at a pH between 8.0 and 8.8. Drawing on similar approaches, such as Yang et al. (2021), who stabilized saliva samples for the RT-LAMP detection of SARS-CoV-2, we employed NaOH to adjust the pH of chicken samples to a range of 7.3–7.7 [18].

Key parameters, including NaOH concentration, meat cut type, solution volume, and homogenization/incubation time, were systematically optimized. Pretreatment was applied to 50 chicken breast meat samples, resulting in pH values falling predominantly within the desired range, except for two samples with borderline acceptable values (pH 7.27 and 7.28). Representative samples from the first (Q1) and third quartiles (Q3) were chosen for downstream analysis.

To assess the efficiency of the cLAMP assay with and without DNA extraction, pretreated samples underwent both DNA extraction and a simplified heat-treatment protocol. For extracted DNA, amplifications were successful for both samples, with a detectable threshold of 10 CFU/µL for Sample A and 1 CFU/µL for Sample B. These results suggest that the assay is capable of detecting extremely low levels of *Salmonella*.

Heat treatment, while less sensitive than DNA extraction, also produced promising results, with successful amplification of the undiluted template and select dilutions for both samples. This disparity in sensitivity could stem from the adsorption of DNA onto cellular organelles or insufficient disruption during heat treatment. Additional cycles of heat treatment may improve DNA yield. Nevertheless, the results confirmed that heat treatment, coupled with sample pretreatment, is a viable and cost-effective alternative to DNA extraction for field applications.

Previous studies have been published that focus on detecting *Salmonella* without employing either DNA extraction or enrichment steps. For example, Kim et al. (2023) developed a LAMP-based *Salmonella* detection assay that did not include an extraction step. This assay could detect 10 CFU/mL after 30 min of amplification at 65 °C and an initial concentration of 3 CFU/g in artificially contaminated chicken samples following a 60 min enrichment step. Another study by Wu et al. (2015) aimed to develop a detection assay without an enrichment step but including a DNA extraction step. They detected up to 4 CFU/g in *Salmonella*-contaminated lettuce, completing the assay in 3 h [19,20].

The heat-treatment approach, while slightly less sensitive than DNA extraction, remains a practical and efficient method for detecting *Salmonella* in resource-limited environments. This study highlights the potential of the cLAMP assay as a rapid, cost-effective diagnostic tool with specificity for *Salmonella* species and sensitivity comparable to that of real-time PCR.

## 5. Conclusions

Regardless of the approach used to extract the genetic material from the spiked samples, we successfully detected Salmonella spp. using the cLAMP without any pre-enrichment steps. To the best of our knowledge, no prior study has used a cLAMP approach mediating an in-house buffer with Phenol Red for Salmonella spp. detection, without relying on enrichment and DNA extraction.

Although this assay was conducted under ideal conditions, our study provides tangible evidence of the feasibility of running a LAMP assay with minimal prior handling and opens up the possibility of applying this approach to other sample matrices and detecting other foodborne pathogens. Future work will include validation on larger sample sizes, to better align with regulatory standards and assess the method’s scalability for heterogeneous pathogen distribution.

## Figures and Tables

**Table 1:** *hilA* primer set.

Primer	Sequence	Length (bp)
Forward outer (F3)	5′-GCTACGCTCAGAAAAGAAAGTC-3′	22
Backward outer (B3)	5′-GTAACTTTGTGACATGCCTGTC-3′	22
Forward inner (FIP)	5′-GAATATGCCGTTCTGGTCATCC TTTGTGGAATGACCTGGTCCATACC-3′	47
Backward inner (BIP)	5′-ACAGCCTTCTATTTCTCGTAGCGC GACGCGGAAGTTAACGAAGA-3′	44
Loop forward (LF)	5′-CGGCCGCTCTAACACTCATT-3′	22
Loop backward (LB)	5′-CGCTGTATTTATGCCTTACGACG-3′	23

**Table 2:** NaOH concentration used in the pretreatment trials of chicken meat samples presented with the corresponding pH values.

NaoH Concentration	6 mM	7.5 mM	10 mM	12.5 mM	13 mM	15 mM
pH range	6.4–5.7	6.8–6.2	7.5–6.9	7.7–7.2	8–7.5	8.3–7.8

## Data Availability

The original contributions presented in the study are included in the article, further inquiries can be directed to the corresponding author.
